# Technology Developments in Structural Health Monitoring and Integrity Maintenance

**DOI:** 10.1155/2014/976927

**Published:** 2014-12-31

**Authors:** Ting-Hua Yi, Ying Lei, Hua-Peng Chen, Xiao-Wei Ye, Fei Kang, Hao Wang

**Affiliations:** ^1^School of Civil Engineering, Dalian University of Technology, Dalian 116023, China; ^2^School of Architecture and Civil Engineering, Xiamen University, Xiamen 361005, China; ^3^Medway School of Engineering, The University of Greenwich, Kent ME4 4TB, UK; ^4^Department of Civil Engineering, Zhejiang University, Hangzhou 310058, China; ^5^School of Civil Engineering, Purdue University, West Lafayette, IN 47907, USA; ^6^School of Civil Engineering, Southeast University, Nanjing 210096, China

Structural health monitoring (SHM) has gained a significant number of attentions from the engineering communities in the past two decades, which integrates the knowledge of a variety of disciplines including structural engineering, material science, computer science, signal processing, and data management [1–3]. A main purpose of the development of an SHM system is deemed to facilitate the routine inspection and maintenance activities of the targeted engineering infrastructures. With the aid of an instrumented long-term SHM system, the structural behavior and safety performance of the structure can be promptly evaluated by use of the huge amount of measurement data, and the optimal maintenance schedules can be executed by the infrastructure managers [4]. It includes the connectivity and information exchange between the participating institutions and individual members, the awareness of the SHM and integrity maintenance disciplines and tools among end users, and essential reference materials for the situations where a ranking of structures to be rehabilitated is necessary because of insufficient budget available.

Therefore, in the light of these considerations, this special issue was launched in this journal, an SCI-indexed international journal. The papers in this special issue present the most recent advances, progress, and ideas in the field of the SHM and integrity maintenance and its application. It includes smart, bioinspired, nanometer, wireless, and remote sensing technology, sensor placement and optimization strategies, data compression, cleaning, mining and fusing technology, pattern recognition, feature extraction and damage detection and assessment, design, retrofit, maintenance, renewal and risk management of civil infrastructure, and application of SHM for heritage structures, historical monuments, and old bridges.

After two-round peer reviewing, totally 55 research articles are received and 28 out of them are finally accepted for publication in this special issue. Among them, four papers are review articles. The paper “*Structural health monitoring of civil infrastructure using optical fiber sensing technology: a comprehensive review*” by X. W. Ye et al. presents a summary of the basic principles of various optical fiber sensors, innovation in sensing and computational methodologies, development of novel optical fiber sensors, and the practical application status of the optical fiber sensing technology in civil infrastructure monitoring. The paper “*Dynamic responses and vibration control of the transmission tower-line system: a state-of-the-art review*” by B. Chen et al. reviews the dynamic responses and vibration control of the transmission tower line system as well as the disaster monitoring and mitigation of the system subjected to dynamic excitations. The paper “*Recent research and applications of numerical simulation for dynamic response of long-span bridges subjected to multiple loads*” by Z. Chen and B. Chen addresses the key issues involved in dynamic response analysis of long-span multiload bridges based on numerical simulation technologies and the engineering applications of newly developed numerical simulation technologies for safety assessment of long-span bridges. The paper “*A review on strengthening steel beams using FRP under fatigue*” by M. Kamruzzaman et al. summarizes the existing FRP reinforcing techniques for fatigue damaged structural steel elements.

The following papers address the research work on structural damage detection. The paper “*Damage identification for large span structure based on multiscale inputs to artificial neural networks*” by W. Lu et al. proposes a structural damage identification method by combining the measured results from strain sensors and accelerometers in the noisy environment based on the artificial neural network. The paper “*Damage assessment of two-way bending RC slabs subjected to blast loadings*” by H. Jia et al. investigates the blast response and damage assessment of a two-way bending RC slab subjected to blast loadings. The paper “*Structural damage identification based on rough sets and artificial neural network*” by C. Liu et al. conducts the research on potential applications of the rough sets theory and the artificial neural network method for structural damage detection. The paper “*Damage detection on sudden stiffness reduction based on discrete wavelet transform*” by B. Chen et al. presents the damage detection on sudden stiffness reduction of building structures based on the discrete wavelet transform. The paper “*Damage detection of structures identified with deterministic-stochastic models using seismic data*” by M.-C. Huang et al. addresses a deterministic-stochastic subspace method for damage identification which has been experimentally verified to detect the equivalent single-input-multiple-output system parameters of the discrete time state equation.

Other papers focus on the research related to the development of integrated SHM systems and novel sensing technologies. The paper “*Integrated system of structural health monitoring and intelligent management for a cable-stayed bridge*” by B. Chen et al. describes the integrated system for structural monitoring and intelligent management of the cable-stayed Zhijiang Bridge, China. The paper “*Full-scale measurements and system identification on Sutong Cable-stayed Bridge during typhoon Fung-Wong*” by H. Wang et al. analyzes the wind data and the structural vibration responses obtained from the SHM system installed on the cable-stayed Sutong Bridge, China, during a typhoon. The paper “*Study on typhoon characteristic based on bridge health monitoring system*” by X. Wang et al. investigates the typhoon characteristics by use of the measured data from the bridge health monitoring system instrumented on the Jiubao Bridge, China. The paper “*Numerical simulation of monitoring corrosion in reinforced concrete based on ultrasonic guided waves*” by Z. Zheng et al. predicts the location of the pitting corrosion in reinforced concrete based on the ultrasonic guided waves. The paper “*Study on dynamic response measurement of the submarine pipeline by full-term FBG sensors*” by J. Zhou et al. measures the dynamic responses of the submarine pipeline by use of the FBG sensing technology. The paper “*Case study on the maintenance of a construction monitoring using USN-based data acquisition*” by S. Kim et al. develops a ubiquitous sensor network for monitoring and maintenance of the building structure. The paper “*In-line ultrasonic monitoring for sediments stuck on inner wall of a polyvinyl chloride pipe*” by H. Seo et al. verifies the applicability and effectiveness of the ultrasonic monitoring of sediments stuck on the inner wall of polyvinyl chloride pipes.

The subsequent papers present the investigations on the structural performance under seismic excitations. The paper “*Numerical simulation on slabs dislocation of Zipingpu concrete faced rockfill dam during the Wenchuan earthquake based on a generalized plasticity model*” by B. Xu et al. investigates the slab dislocation phenomenon of the Zipingpu concrete faced rockfill dam during earthquake. The paper “*An improved multidimensional MPA procedure for bidirectional earthquake excitations*” by F. Wang et al. develops an improved multidimensional modal pushover analysis method for estimating the response demands of structures subjected to bidirectional earthquake excitations. The paper “*A methodology for multihazards load combinations of earthquake and heavy trucks for bridges*” by D. Sun et al. presents a modified model considering the advantages of Ferry Borges-Castanheta's model and Turkstra's rule in converting the random process into the random variables for earthquake analysis. The paper “*Experimental study of the seismic performance of L-shaped columns with 500 MPa steel bars*” by T. Wang et al. addresses the experimental results of six L-shaped RC columns with 500 MPa steel bars for seismic performance assessment.

The remaining papers in this special issue introduce the research outcomes on monitoring of hydraulic and geotechnical structures. The paper “*Effects of outlets on cracking risk and integral stability of super-high arch dams*” by P. Lin et al. presents the outlet cracking in the Goupitan and Xiaowan arch dams by use of the nonlinear finite element method. The paper “*Real-time safety risk assessment based on a real-time location system for hydropower construction sites*” by H. Jiang et al. proposes a method for real-time safety risk assessment for the hydropower construction site. The paper “*Ant colony optimization analysis on overall stability of high arch dam basis of field monitoring*” by P. Lin et al. conducts a dam ant colony optimization analysis of the overall stability of the high arch dam on the complicated foundation. The paper “*Displacement back analysis for a high slope of the Dagangshan hydroelectric power station based on BP neural network and particle swarm optimization*” by Z. Liang et al. presents a displacement back analysis for the slope using an artificial neural network model and particle swarm optimization model. The paper “*Uplifting behavior of shallow buried pipe in liquefiable soil by dynamic centrifuge test*” by B. Huang et al. carries out the dynamic centrifuge model test to investigate the uplifting behavior of the shallow buried pipeline subjected to seismic vibration in the liquefied site.
